# Hollow nanostructures of metal oxides as next generation electrode materials for supercapacitors

**DOI:** 10.1038/s41598-018-19815-y

**Published:** 2018-01-22

**Authors:** Vikas Sharma, Inderjeet Singh, Amreesh Chandra

**Affiliations:** 1 School of Nanoscience and Technology, Indian Institute of Technology Kharagpur, Kharagpur, 721302 West Bengal India; 2 Department of Physics, Indian Institute of Technology Kharagpur, Kharagpur, 721302 West Bengal India

## Abstract

Hollow nanostructures of copper oxides help to stabilize appreciably higher electrochemical characteristics than their solid counter parts of various morphologies. The specific capacitance values, calculated using cyclic voltammetry (CV) and charge-discharge (CD) studies, are found to be much higher than the values reported in literature for copper oxide particles showing  intriguing morphologies or even composites with trendy systems like CNTs, rGO, graphene, etc. The proposed cost-effective synthesis route makes these materials industrially viable for application in alternative energy storage devices. The improved electrochemical response can be attributed to effective access to the higher number of redox sites that become available on the surface, as well as in the cavity of the hollow particles. The ion transport channels also facilitate efficient de-intercalation, which results in the enhancement of cyclability and Coulombic efficiency. The charge storage mechanism in copper oxide structures is also proposed in the paper.

## Introduction

Till a decade or two back, fossil fuel based sources were expected to meet the increasing demand for energy owing to rapid urbanization. Then came the *evidences*, proving the limited reserves of fossil fuels and their long-term devastating impact on the climate. Consequently, worldwide research saw a paradigm shift and renewed vigor towards finding socio-economical renewable energy sources for grid and off-grid applications^1–3^. Initially, solar and fuel cells attained prominence before other renewable energy sources such as wind, tidal, bio-, etc. became competitive^4–8^. To ensure energy security and tackle the intrinsic limitation of “intermittent availability”, two storage technologies, which have remained most investigated and/or used are: Li-batteries and supercapacitors^9–13^. Supercapacitors are also considered as viable shield to save the expensive Li-batteries from transients or shocks, associated with sudden change in demand or supply^14^. The long cycle life (>few tens of thousands) has also made supercapacitors attractive for applications in automobiles, electric locomotives, smart phones, soldier shoes, sensors, load leveler, etc^15–17^.

Based on the charge storage mechanism, supercapacitors are broadly classified as electric double layer capacitor (EDLC) and pseudocapacitors^18^. Initially, EDLCs were fabricated using several forms of carbon such as: activated carbon, CNTs, graphene-like, graphite, etc^19–22^. The low specific capacitance, energy density and tedious processes involved to achieve high surface area active material(s) have mostly led to saturation in the device performance. Consequently, transition metal oxides (TMOs) based pseudo-capacitors started to overshadow EDLCs even though the cost or environmental impact were higher^23^. In comparison, pseudo-capacitors fabricated using various metal-oxides, hydro-oxides or conducting polymers generally exhibit much higher specific capacitances than EDLCs. Knowing the fact that the pseudocapacitor’s capacitance is driven by the faradaic redox reactions at the material surface, their performance can also be increased by incorporating redox additives into the electrolyte^24^.

Mostly, the strategy of using various intriguing morphologies of a metal oxide to tune the deliverable specific capacitance is employed. These include nanoflowers, nano-ribbons, core-shell, etc^25–27^. Once the power density obtained using a particular solid metal oxide with various morphologies indicate saturation, attempts start to investigate the next metal oxide. This is somehow leading to duplication and/ or monotonous approaches, without achieving the ultimate target of bringing quantum jump in the specific capacitance while reducing the carbon footprint^28–30^.

RuO_2_ was amongst the first metal oxides, which brought pseudo-capacitors to the forefront, as very high specific capacitance values could be obtained^31^. Very soon, its high cost and toxicity forced researchers to look for alternatives. Consequently, less expensive and environmentally compatible transition metal oxides (TMOs) have become industrially acceptable, as the cost factor and climate mitigation laws are two major concerns for the manufacturers. The alternative TMOs include MnO_2_, V_2_O_5_, SnO_2_, Fe_2_O_3_, Co_3_O_4_, etc^32–37^. Another simpler TMO (with much higher functionality, useful for both - batteries and supercapacitors), which has been investigated, is copper oxide (Cu_2_O and CuO). Its particle with many interesting morphologies such as nano/microcubes, nanocage, nano-flower, etc. have been employed^38,39^. Out of two copper oxides, CuO has been more widely used. However, Cu_2_O remains an ignored member of copper based oxide family even though it has a theoretical specific capacitance of 2250 F g^−1^ and significant faradaic response^40–42^.

In this paper, using these two conventional and simple oxides of copper viz., Cu_2_O and CuO, it is shown that a hitherto ignored strategy of using hollow nanostructures can bring quantum jump in the electrochemical performance of TMOs. These materials, owing to low mass and high electrochemical response, may also help to reduce the weight of the final device, a factor extremely critical for most industries. Till date, hollow nanostructures have mostly been thought to be more useful for applications such as catalysis, sensors, solar cells, nanoscale chemical reactors, drug delivery, etc^43–47^. Low diffusion length and high ionic percolation associated with rough and porous surface boost the EDLC behavior. It is shown that copper (I) oxide hollow structures return nearly 70–80% higher specific capacitance than the corresponding solid particles. The discussed Cu_2_O hollow structures also augments cyclic stability and can serve as pure negative electrode material in a wide potential window. The results, when compared with relevant literature, suggest that forming composites with other expensive components such as graphene, reduced graphite oxide, etc. may not be required, if the hollow particles are carefully tuned and used as active electrode materials.

## Results and Discussions

### Characterization and growth mechanisms

X-ray diffraction (XRD) clearly showed the formation of pure Cu_2_O with cubic (*Pn* $\bar{3}$ *m*) crystal structure. No impurity peaks were observed in both the XRD profiles and corresponding indexing of the X-ray diffractograms is given in Fig. S1 of the supporting evidence. XRD profile of CuO hollow nanostructures was similar to those reported earlier^43^. Energy dispersive Analysis of X-Rays (EDAX) spectra were taken for both Cu_2_O hollow nanostructures (Cu(HN))/Cu_2_O octahedrons (Cu(OCT)) and the related results are discussed in detail in the supplementary information. (Fig. S2(a,b) and Table S1). The data confirmed the expected concentration of the constituents.

**Figure 1:**
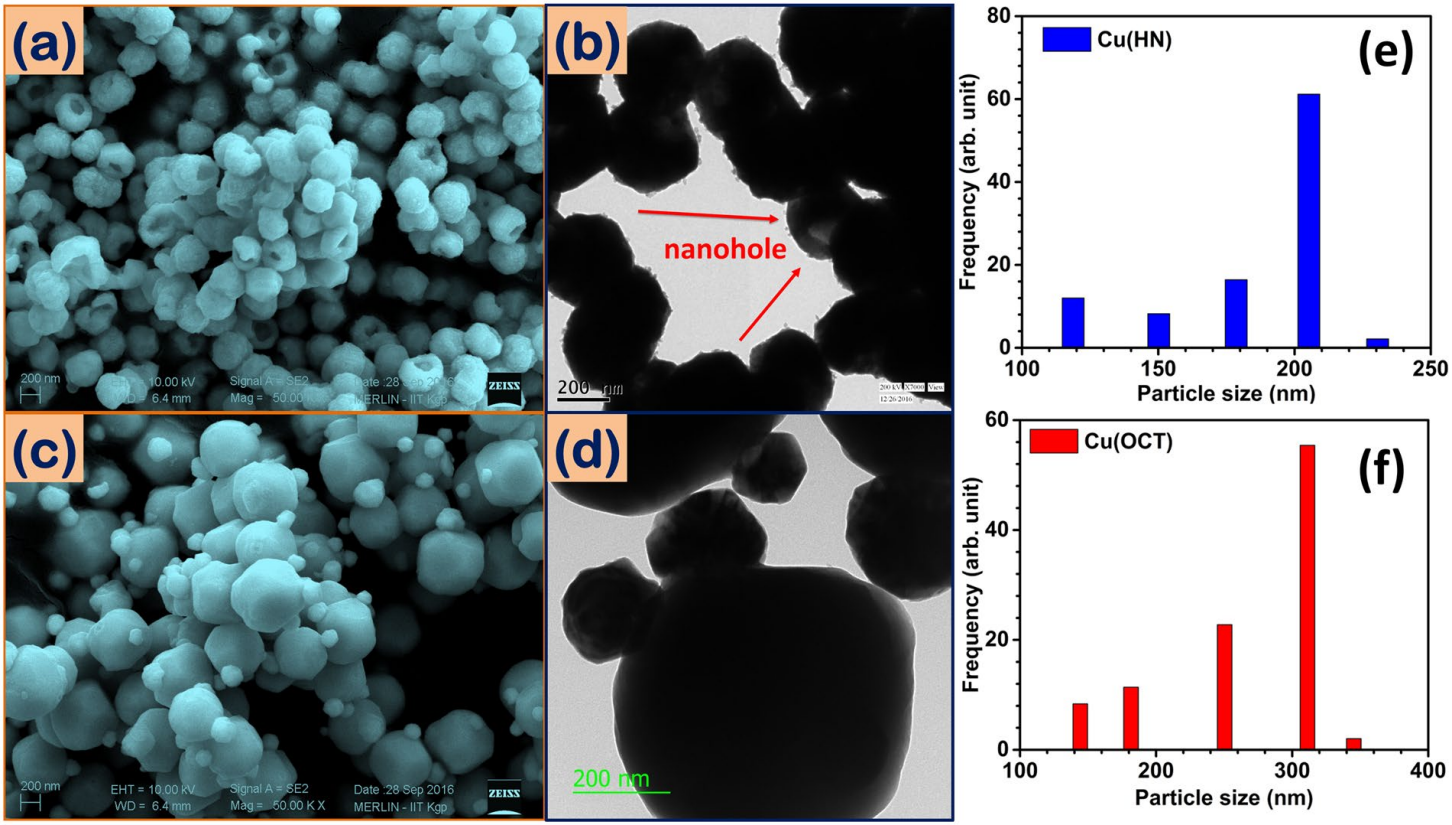
(**a**,**c**) SEM, (**b**,**d**) TEM images and (**e**,**f**) particle size distribution of Cu(HN) and Cu(OCT), respectively.

It is now well accepted that the electrochemical behavior of the TMOs is strongly affected by the particle morphologies. Figure 1a–d, show the scanning electron microscopy (SEM) and transmission electron microscopy (TEM) micrographs, which clearly indicated the formation of two morphologies (hollow and solid) of Cu_2_O. Nearly uniform sized hollow nano-spheres, with diameter of ~200–300 nm and cavity dimension of ~ 150 nm were discernible (Fig. 1a,b). The hollow nanospheres had rough exterior shell. As the precursor concentration was carefully varied, solid Cu(OCT) could be obtained. These had smooth and curved corners with longest axis of ~400 nm. Some smaller octahedrons were also present, which could be attributed to the nucleation, growth and subsequent Ostwald type process, that is discussed later (Fig. 1c,d). Different area TEM micrographs are also shown in the supporting information regarding the Cu(HN), which further confirmed the uniform distribution of hollow particles (see Fig. S3(a–f)). Confirmation of CuO hollow nanostructures can be done from the detailed explanation by Singh *et al*.^43^. Particle size distributions in the Cu_2_O samples used in the present studies are given in Fig. 1e,f. N_2_ adsorption-desorption isotherm is also depicted in Fig. S4. The Brunauer–Emmett–Teller (BET)analysis showed that the effective surface area of Cu(HN) (~10 m^2^/g) was more than two times higher than that of Cu(OCT) (~4 m^2^/g). The significance of this observation is also presented in the supporting evidence. Fig. S5 shows the particle size distribution for CuO hollow nanostructures.

Transformation from hollow (HN) to solid (OCT) type Cu_2_O can be explained as a convoluted phenomenon of thermodynamically driven nucleation and Ostwald ripening type growth mechanism. In the present case, the growth of the particles seems to be a convoluted picture of two mechanisms viz., diffusion and aggregation. In the growth of the overall crystal structure, growth of every single facet is important. According to Gibbs- Wulff energy minimization theory, every crystal organizes itself in the state of lowest surface Gibbs free energy. Moreover, according to Bravais law, all crystal faces develop along the planes defined by the points in a particular lattice. Combining both the laws, the growth of the crystal takes place along the various crystal planes which leads to the formation of morphology that has the minimum overall surface Gibbs free energy^48^. This would vary as the concentration of precipitating agent i.e. NaOH will change because the rate of nucleation is strongly dependent on the supersaturation state.

**Figure 2:**
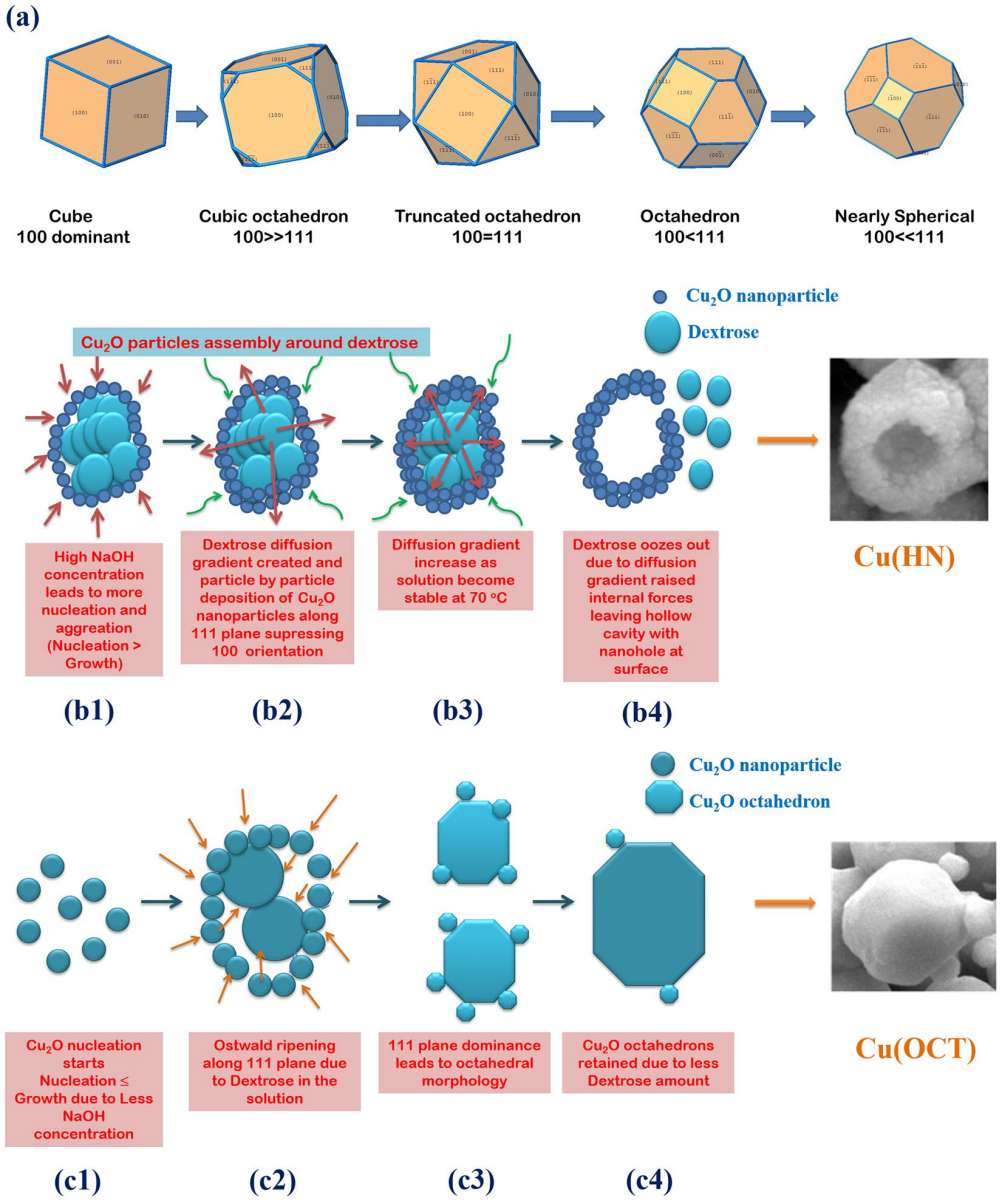
(**a**) Artificial crystal morphologies of Cu_2_O crystal explaining growth of Cu(HN) and Cu(OCT), (b1-b4) Growth mechanism Cu(HN) and (c1-c4) Cu(OCT).

In case of cuprites like Cu_2_O, the growth is preferred along the crystallographic [100] and [111] planes. But the overall surface energy of 111 plane is lower than 100 plane (111 < 100 < 110). Therefore, the crystal unit cell tries to transform from 100 unstable plane to 111stable plane^49^. Figure 2a shows the evolution of 111 plane from 100 plane, owing to the increase in the growth-determining factor (GDF). Higher concentrations of GDF, leads to the stabilization of nearly spherical morphology, due to the refinement of edges generated by the (111) plane (making them curved rather than sharp). At lower concentrations, morphology terminates at any of the earlier step. In the present case, during the formation of Cu(HN), the concentration of NaOH was much higher in comparison to the synthesis protocol for Cu(OCT). So, the rate of nucleation is expected to be higher in the case of hollow nanospheres. Therefore, the evolution of particle morphologies can be expected as under:

(i) Due to the higher concentration of NaOH, rate of nucleation (ϒ_N_) > rate of growth (ϒ_G_). Precipitation of Cu(OH)_2_ nanoparticles starts. These nanoparticles form aggregates at the interface of dextrose molecules and the solution. Along with this, presence of the reducing agent i.e., dextrose, in high concentration, will enforce the peeling of Cu(OH)_2_ consistently, both from inside/outside and stabilization of aggregated nanoparticles in thermodynamically preferable 111 planer orientation, as seen in Fig. 2b1–b4.
(ii) With time, a concentration gradient of dextrose can be expected that would vary the extent of peeling. Near the area of high concentration, a cavity would form, which would be forced to grow further because the fluidic reducing agent will get entrapped in it. This would lead to formation of hollow particles, which are shown in Fig. 2b4.
(iii) In the case of Cu(OCT), the concentration of NaOH was low, which would lead to the scenario where ϒ_N_ ≤ ϒ_G_. Therefore, the particle growth would be predominantly controlled by the diffusion process.
(iv) Finally, due to the presence of dextrose, the particles will be isotropically reduced from all sides with much lesser probability of cavity formation. The preferred growth would be along [100] with subsequent evolution along [111] direction. With increasing reaction time, Ostwald type ripening will occur and larger sized particles will stabilize (see Fig. 2c1–c4).

The growth mechanism for CuO nanostructures was already discussed in detail by Inderjeet *et al*.^43^.

### Electrochemical studies

**Figure 3:**
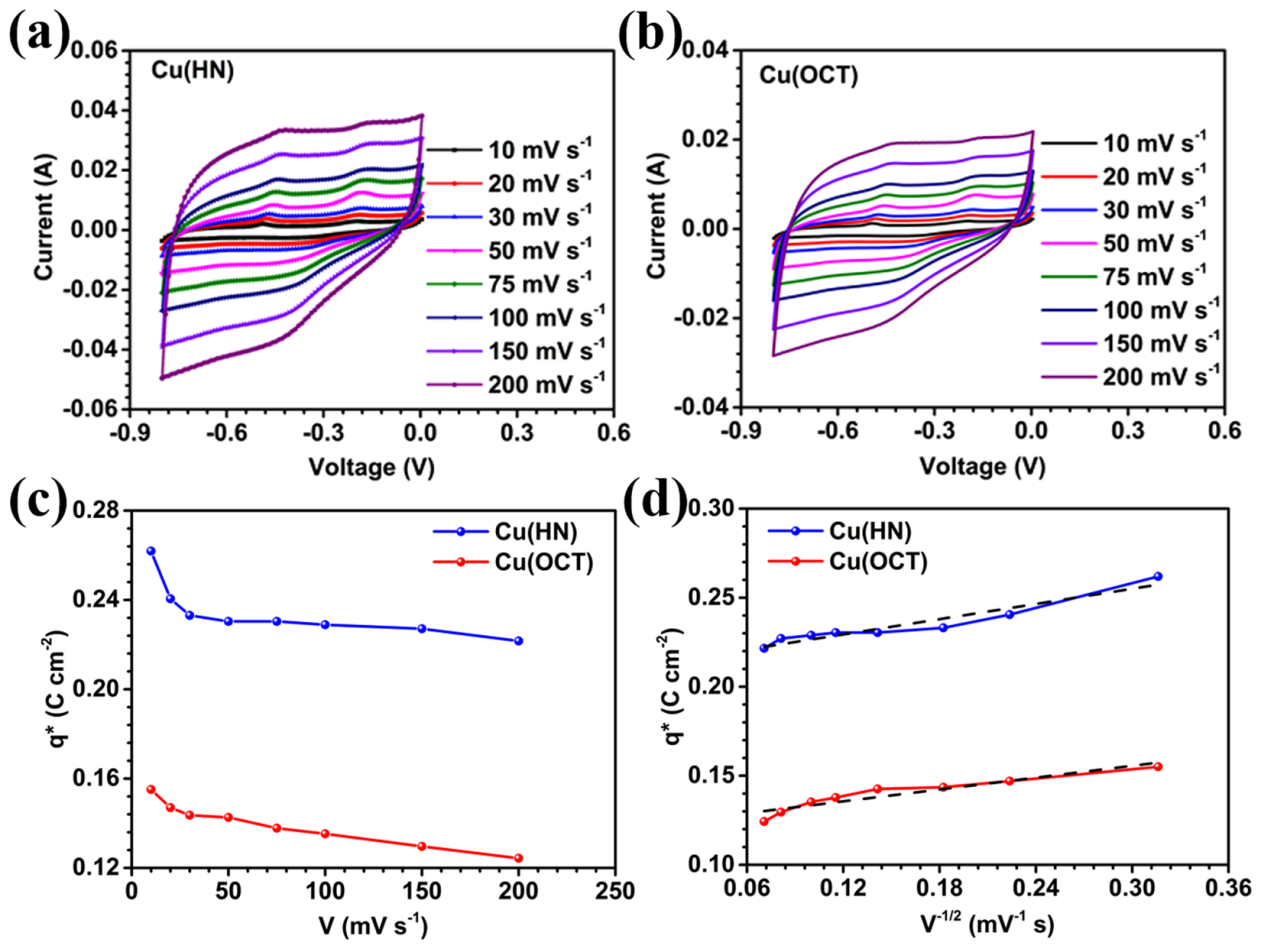
CV profiles of (**a**) Cu(HN), (**b**) Cu(OCT) at different scan rates, (**c**) Voltammetric charge at different scan rates for Cu(HN) and Cu(OCT) and (**d**) voltammetric charge q* plot as a function of V^−1/2^, where V is scan rate. The double layer charge (q_0_) is determined at the V^−1/2^ = 0 corresponding to the intercept of the fitting line.

It is clear from the previous sections that two very different morphologies of copper oxides can be stabilized. The importance of one morphology over other can only be claimed provided it shows significant improvement in physical properties. This section will show appreciable improvement in the electrochemical performance that can be achieved using hollow structures. While determining the electrochemical performance using a three-electrode assembly, mass of the active material was kept same for both Cu(HN) and Cu(OCT) viz., 1 mg cm^−2^. Figure 3a,b shows the evolution of CV profiles as a function of scan rates. The closed CV curves were similar to those expected in materials, which show predominant pseudocapacitive behavior^50^. The comparative study proved beyond doubt that the hollow structures had much high electrochemical behavior. The electrochemical response in hollow structures would be controlled by surface reactions as well as the diffusion of electrolyte ions within the cavity. This will logically be higher in comparison to solid structures, where only the surface reactions will play the dominant role. Zeta potential values also justified the claim of higher number of active surface sites for ion adsorption and intercalation in hollow structures.

**Figure 4:**
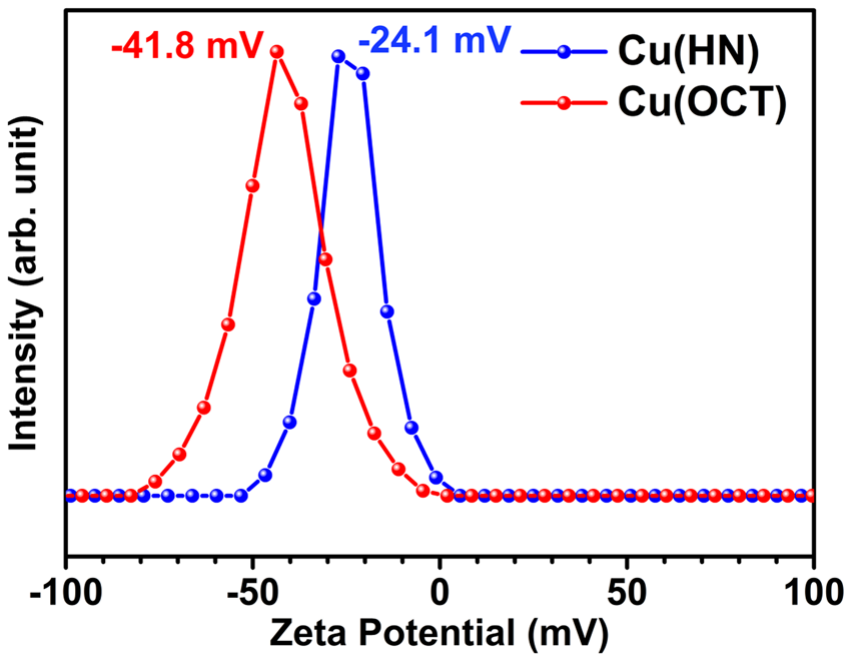
Zeta potential curves for Cu(HN) and Cu(OCT).

Cu_2_O is known to show negative zeta potential^51^. Typical Zeta potential curves for copper oxide samples are shown in Fig. 4. During chemical reaction, electropositivity of the surface is determined by the overall pH of the solution. As the material gets dispersed, the oppositely charged ions i.e., OH^−^ ions in de-ionized (DI) water, would get attracted towards the surface and form a layer^52^. On the other hand, the released H^+^ ions reduce the pH of the solution and zeta potential shifts towards the positive regime. From Fig. 4, zeta potential values for Cu(HN) and Cu(OCT) were estimated as −24.1 mV and −41.8 mV, respectively. Hollow nanostructures returned higher electropositivity, which can only happen when there is increased volume to facilitate OH^−^ accommodation. This qualitative estimation was found to be in accordance with the inferences drawn using CV profiles, which showed the hollow structures had higher electrochemical response.

CV profiles for both Cu_2_O based materials show two anodic peaks at −0.4 V and −0.15 V, as in Fig. 3a,b. These peaks could be attributed to the formation of CuO and Cu(OH)_2_.One broad cathodic peak (having two smaller peaks) at −0.45 V was related with the reformation of Cu_2_O from CuO and Cu(OH)_2_. Chemically, the anodic and cathodic reactions could be represented as^50^:

1 $$\frac{1}{2}{\boldsymbol{C}}{{\boldsymbol{u}}}_{2}{\boldsymbol{O}}+{\boldsymbol{O}}{{\boldsymbol{H}}}^{{\boldsymbol{-}}}\leftrightarrow C{\boldsymbol{uO}}+\frac{1}{2}{{\boldsymbol{H}}}_{2}{\boldsymbol{O}}+{{\boldsymbol{e}}}^{-}$$

2 $$\frac{1}{2}{\boldsymbol{C}}{{\boldsymbol{u}}}_{2}{\boldsymbol{O}}+\frac{1}{2}{{\boldsymbol{H}}}_{2}{\boldsymbol{O}}+{\boldsymbol{O}}{{\boldsymbol{H}}}^{-}\leftrightarrow {\boldsymbol{Cu}}{({\boldsymbol{OH}})}_{2}+{{\boldsymbol{e}}}^{-}$$

3 $${\boldsymbol{CuOH}}+{\boldsymbol{O}}{{\boldsymbol{H}}}^{-}\,\leftrightarrow {\boldsymbol{CuO}}+{{\boldsymbol{H}}}_{2}{\boldsymbol{O}}+{{\boldsymbol{e}}}^{-}$$

4 $${\boldsymbol{CuOH}}+{\boldsymbol{O}}{{\boldsymbol{H}}}^{-}\leftrightarrow {\boldsymbol{Cu}}{({\boldsymbol{OH}})}_{2}+{{\boldsymbol{e}}}^{-}$$

It is now believed that estimation of contributing active sites is more important than determination of initial surfaces areas of the materials, which is generally reported. This is because the effective surface area in the final device is much lower than the starting value as the surface of the active material gets coated with polymers like PVDF, etc^53^. To determine the contributing active sites, determination of voltammetric charge of the material is critical. This is directly related to the available surface area of the coated material on the electrode. The voltammetric charge (q*) is obtained using the following relation^54^:

5 $${{\boldsymbol{q}}}^{\ast }=\frac{\int i\,{\boldsymbol{dE}}\,}{{\boldsymbol{AV}}}$$

where, V, i, E and A denote the scan rate, voltammetric current (mA), potential (mV) and geometric area of the electrode (cm^2^), respectively. In addition, q* can also be calculated by using the relation^54^:

6 $${{\boldsymbol{q}}}^{\ast }={{\boldsymbol{q}}}_{0}+{\boldsymbol{k}}{{\boldsymbol{V}}}^{-1/2}$$

where, q_0_ is the charge contribution by double layer. This approach can also be employed to extract information pertaining to the relative surface area of the coated portion on the electrode.

Figure 3c shows the q* vs V curve for Cu(HN) and Cu(OCT). The curve clearly indicated towards consistently decreasing adsorbed charges on the surface of both the electrodes, with increasing scan rate. This behavior has also been observed in systems based on other metal oxides with pseudocapacitive behavior like RuO_2_, IrO_2_, etc^54–56^. Higher interaction of ions with active sites of the electrode material leads to enhanced adsorption of charges on the electrode surface. This would result is faradaic response along with the formation of double layer capacitance. Therefore, at lower scan rates, redox peaks would become prominent, before diminishing at higher scan rates. In the present case, the adsorbed charge was ~0.27 C cm^−2^ at 10 mV s^−1^ for the Cu(HN) which was nearly double the value obtained for Cu(OCT) i.e., ~0.15 C cm^−2^.

**Figure 5:**
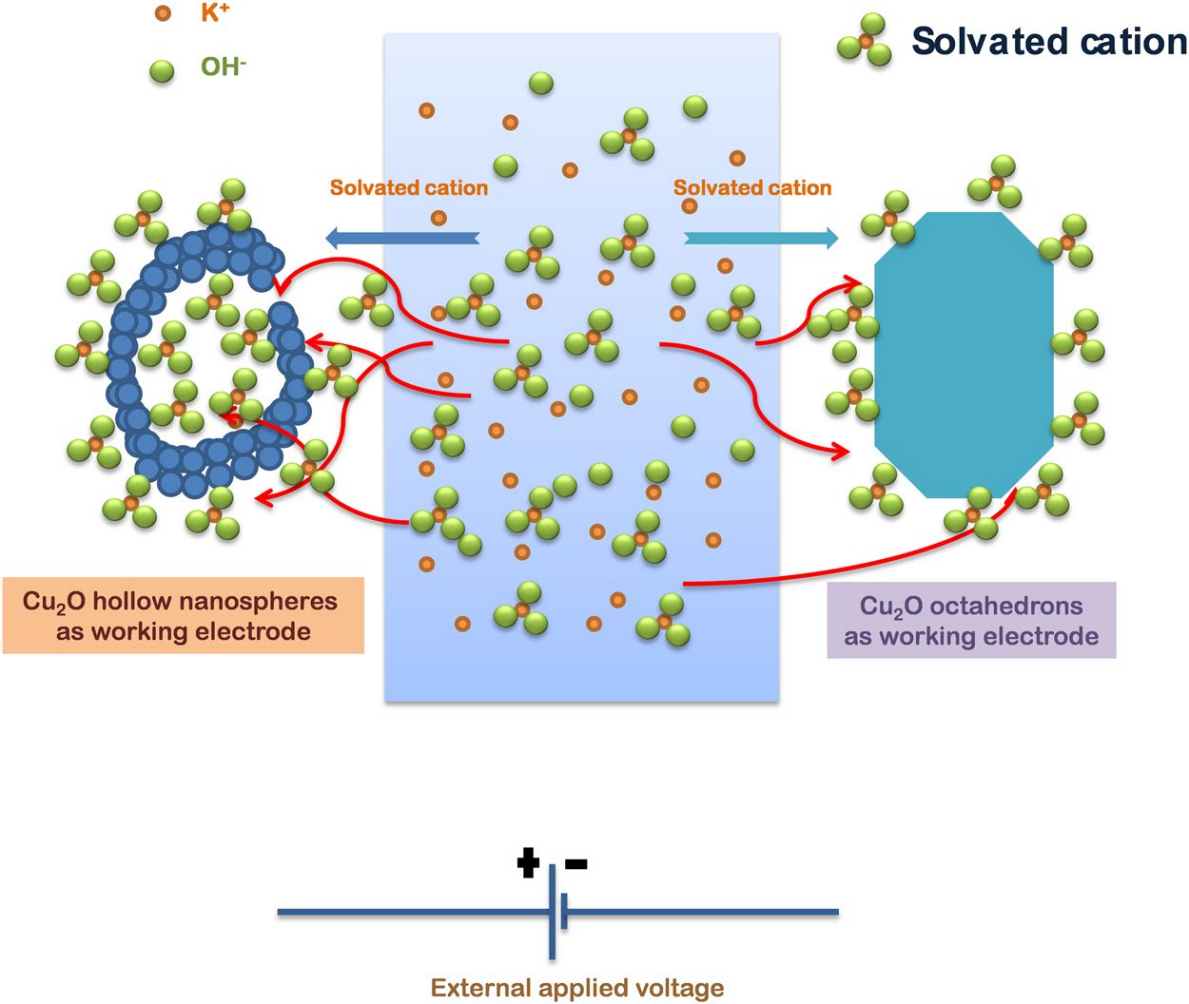
Schematic scheme of the ion interaction with electrode for Cu(HN) and Cu(OCT) as electrode materials.

Figure 3d shows the curve between q* and V^−1/2^ and the intercept of the curve gives the value of q_0_. The value of q_0_ was ~0.22 mC cm^−2^ and ~0.13 mC cm^−2^ for Cu(HN) and Cu(OCT), respectively. Similarly, the surface charge contribution for hollow nanospheres was found to be 1.7 times greater than that obtained for Cu(OCT). This corroborated the inferences drawn using the zeta potential measurements and CV profiles, that the charges present at the electrode surface are more in case of Cu(HN). The increased charge collection at the electrode-electrolyte interface will increase the EDLC driven specific capacitance (enforce higher area under the CV curve). The above results clearly showed that increased surface charge adsorption capacity, owing to higher number of active sites, enhances the electrochemical response. Based on the above discussions, the charge storage mechanism in hollow structures can be schematically explained using Fig. 5.

The specific capacitance values were estimated using the relation:

7 $${{\boldsymbol{C}}}_{{\boldsymbol{CV}}}=\frac{1}{2{\boldsymbol{M}}{{\boldsymbol{S}}}_{{\boldsymbol{r}}}\bigtriangleup {\boldsymbol{V}}}{\int }_{-{\boldsymbol{V}}}^{+{\boldsymbol{V}}}{\boldsymbol{I}}.{\boldsymbol{dV}}$$

where M, S_r_, ΔV and ${\int }_{-V}^{+V}I.dV$ represents the mass of the active material, scan rate, potential window and absolute area under the CV curve, respectively. The values of the specific capacitance as a function of varying scan rates are listed in Table S2. The maximum specific capacitance values obtained at scan rate of 10 mV s^−1^, using hollow spheres (164 F g^−1^) were nearly 70% higher than that observed using solid octahedron particles (97 F g^−1^).

**Figure 6:**
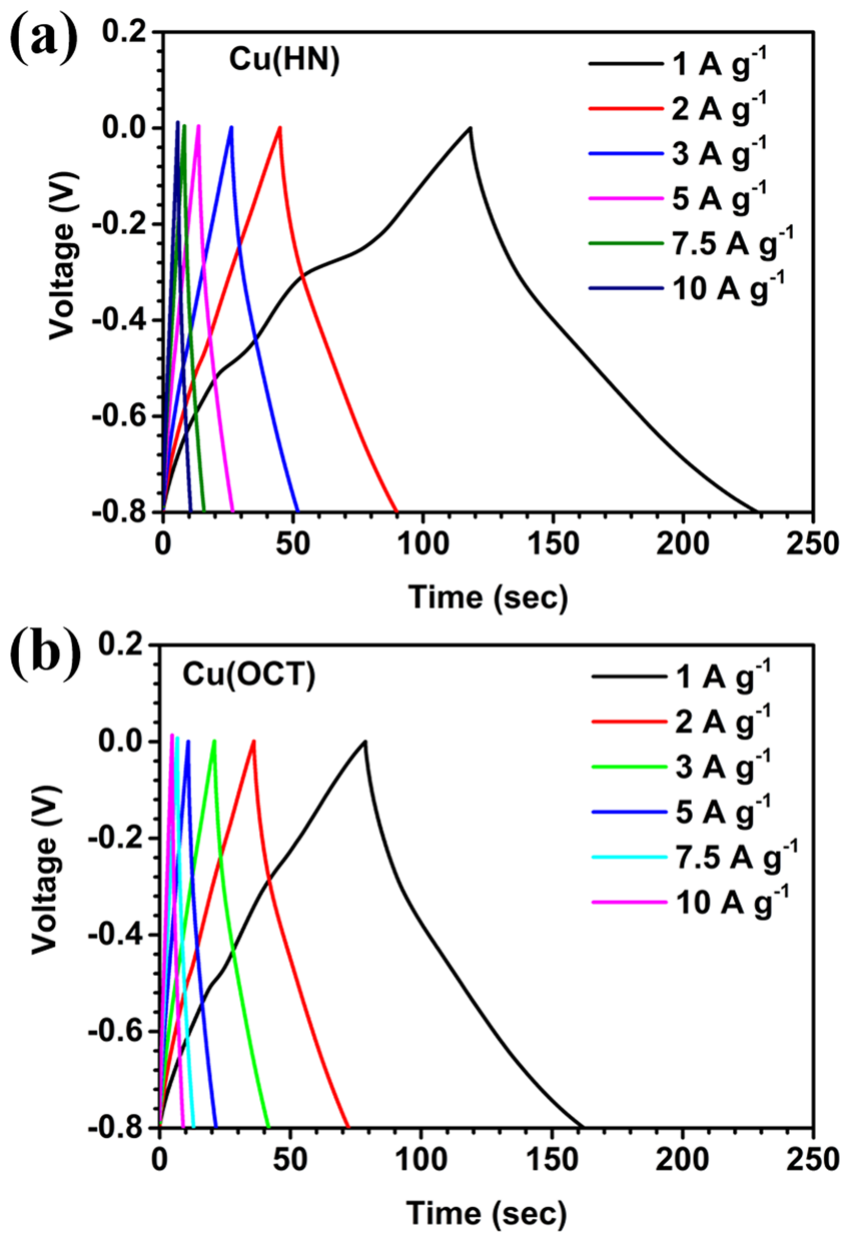
CD curves of (**a**) Cu-HN and (**b**) Cu-OCT at different current densities.

For commercialization of supercapacitors, charge-discharge performance is required, as it provides the quantitative estimation of the deliverable specific capacitance. Figure 6a,b shows the charge discharge profiles of Cu(HN) and Cu(OCT). During the charging cycle, at lower scan rates, two distinguishable regions were evident, which were also distorting the linearity of the profiles. These two portions could be linked to the redox reactions (similar to those visible in the CV profiles)^48^. In a battery material, these redox functions assisted modulations are generally longer and lead to higher energy density. In the present case, at current densities >1 Ag^−1^, the complicated features associated with different redox reaction were considerably suppressed. The charge-discharge profile regained its triangular shape, which reconfirmed the usefulness of Cu_2_O as a supercapacitor electrode material.

Similar type of behavior was observed in both the Cu(HN) and Cu(OCT). On comparing the discharge times of both the materials at different current densities, it was clear that the hollow nanostructures were better suited for ion adsorption and intercalation. The specific capacitance values were calculated by using the relation:

8 $${{\boldsymbol{C}}}_{{\boldsymbol{CD}}}=\frac{{\boldsymbol{I}}.{\boldsymbol{dt}}}{{\boldsymbol{M}}.({\boldsymbol{V}}-{\boldsymbol{IR}})}$$

where I/m, dt, V and IR denote current density, discharge time, operating potential window and voltage drop found at the interface of charging and discharging profile, respectively. The values of specific capacitance were 144/115 F g^−1^ and 105/ 90 F g^−1^ for Cu(HN) and Cu(OCT), respectively, at current density of 1 /2 A g^−1^.

**Figure 7:**
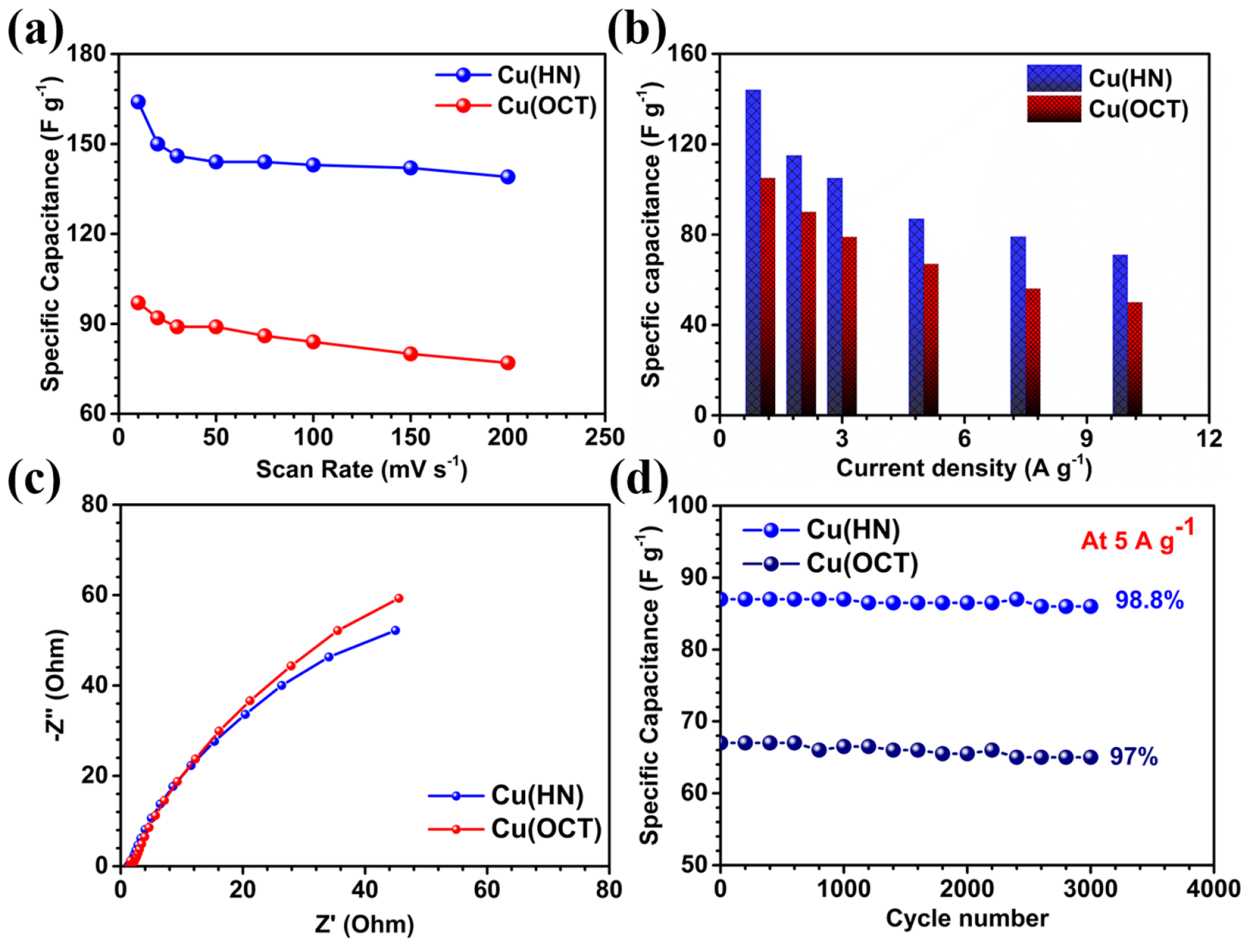
(**a**) Specific capacitance vs scan rate, (**b**) bar graph comparing specific capacitance with current densities, (**c**) EIS plots and (**d**) specific capacitance variation for 3000 subsequent cycles at 5 A g^−1^ for Cu(HN) and Cu(OCT).

Figure 7a shows the variation of specific capacitance values with scan rate. It clearly showed that, increase in the scan rate, leads to reduced specific capacitance. With the increase in scan rate, the faradaic capacitance contribution decreases and EDLC type specific capacitance dominates. This happens because, at high scan rates, the electrolyte ions do not have enough time to intercalate or de-intercalate.

For a worthwhile device application, the capacitance retention determination is critical. It was found to be ~85% and 79% for Cu(HN) and Cu(OCT), respectively (Fig. 7a), with much higher rate capability in the case of Cu(HN). This further showed that the curved surfaces of the hollow nanostructures, formed by the aggregation of nanoparticles were able to provide sufficient channels for directing the ions towards the inner cavity, even at high scan rate of 200 mV s^−1^. But, in case of Cu(OCT), only the exterior smooth surface was available for ions adsorption and desorption. The values of specific capacitances with current densities are listed in Table S3 and a bar graph showing the comparison of the specific capacitances for both the Cu_2_O materials at different current densities is shown in Fig. 7b. The capacitance retentions at 10 A g^−1^ were 49.4% for Cu(HN) and 47.6% for Cu(OCT), respectively.

The charge transport kinetics of the electrode can be studied by electrochemical impedance spectroscopy (EIS) measurements. Using Nyquist plots, information pertaining to electrode-electrolyte interactions, interfacial effects, equivalent series resistance (ESR), etc. can be extracted^10,21,34^. Figure 7c show that the ESR values for Cu(HN) and Cu(OCT) were ~1.26 Ω and ~1.46 Ω, respectively. If a semicircle is present at the higher frequency region, it gives the information regarding charge transfer kinetics. In our case, distorted semicircle was observed in the high frequency zone, which confirmed low charge transfer resistance at the working electrode and electrolyte interface. This further indicated that the ESR value in Cu(HN) was lower than in case of Cu(OCT). From the lower frequency region, typical Warburg type capacitive response was observed in both the morphologies of Cu_2_O. It has been seen that for a flat sheet like electrodes, the Warburg slope remains at 45°and is proportional to 1/CD^1/2^ where ‘C’ and ‘D’ represent concentration of diffusive species and hydrogen diffusion co-efficient, respectively^57^. However, in electrodes based on porous electrodes and the systems, which involve a sequence of reactions this relation does not hold true. At lower frequencies, both the materials showed nearly similar kind of capacitive behavior in which the slope for Cu(HN) is near to 45° and slightly higher for Cu(OCT). This further indicated about the better electrochemical performance Cu(HN) based electrodes. Moreover, it is well known that hollow structures provide shorter diffusion length for electron and ion transport^39^. This leads to lowering of the ESR value of Cu_2_O hollow nanospheres than thesolid octahedrons. This reaffirmed that Cu(HN) was simulating better ion intercalation, adsorption and desorption.

The cyclic stability of the electrode materials was investigated at a current density of 5 A g^−1^. Figure 7d shows that the capacitance retention after 3000 cycles was ~99% and 97% in Cu(HN) and Cu(OCT), respectively. The typical cycling curves for last 10 cycles are shown in Fig. S6(a, b). This further proved that the materials could be used as electrodes of a supercapacitor. The EIS curves indicate slight increase in the ESR values after 3000 cycles, as shown in Fig. S7(a,b). The values of ESR increased from ~1.26 Ω to ~1.84 Ω in Cu(HN) and from ~1.46 to ~2.10 Ω in Cu(OCT). This increase in the ESR values can happen due to electrode degradation as a function of cycling. From the lower frequency regions in Fig. S7(a,b), it is clear that the capacitive behavior was reduced in case of solid structures whereas it remains constant in Cu(HN) without any considerable degradation.

The electrochemical results obtained from Cu(HN) were compared with some of the recent results on Cu_2_O and their composites (Table 1). The advantages of curved surfaces, low diffusion length, high availability of surface adsorption sites due to the hollow cavity and pure crystalline nature proved that Cu(HN) would be better than the solid counterparts, for supercapacitor applications. The electrochemical performance of synthesized CuO hollow nanostructures was also tested (Fig. S8(a–d)and S9(a,b)). The specific capacitance values, at different scan rates and current densities, are listed in Table S4. The results showed similar behavior as observed in case of Cu(HN), with an appreciable increase in the specific capacitance in comparison to solid counter parts. The values are listed in Table 2. There are some reports that deal with the use of other CuO/Cu_2_O-based composites with similar or even higher specific capacitance values^58–60^. These reports claimed that, with the use of other metal oxides or polymers as composite components with CuO/Cu_2_O, higher values can be achieved. But these studies failed to address the aspect cost escalation that would occur due to the use other more expensive components and also the problems of forming stable composites in large scale. In addition, there are also some other reports that have claimed the enhancement in the specific capacitance by adding suitable conducting elements in the bare metal oxides^61–65^. These conducting elements not only increases the cost of the overall device but also needs tedious, time consuming and costly processes to form the composite structures with metal oxides. Our work clearly shows that hollow morphologies of the same metal oxide can enhance the electrochemical properties without the need of any extra additives. This is significant for the industries, as they would prefer to work with simpler, conventional, robust, abundant and cost effective metal oxides.

**Table 1:** Comparison of present electrochemical result with some recent results on Cu_2_O based electrode materials.

Electrode Material	Current density (A g^−1^)	Specific capacitance (F g^−1^)	Reference
Cu_2_O nanoparticle-MWCNT	2.5	132	^69^
Cu_2_O microstructures	0.1	144	^70^
Cu_2_O/CuO/rGO	1	173	^41^
Cu_2_O/rGO	0.1	31	^71^
**Cu**_**2**_ **O hollow nanospheres**	**1.0**	**144**	**Present work**

**Table 2:** Comparison of present electrochemical result with some recent results on CuO based electrode materials.

Electrode Material	Scan rate (mV s^−1^)	Specific capacitance (F g^−1^)	Reference
CuO nanoparticles	10	76	^72^
CuO-PAA thin films	40	30	^73^
CuO nanostructures	5	96	^74^
CuO nanoflower-rGO	5	136	^74^
CuO nanoparticles	10	20	^75^
CuO nanoparticles-rGO	10	80	^75^
**CuO hollow nanostructures**	**10**	**140**	**Present work**

## Conclusions

The results clearly indicate that using hollow nanostructures of copper oxide can bring appreciable jump in the electrochemical performance in supercapacitors. A simple and scalable one step strategy has been established for the synthesis of hollow and solid Cu_2_O nanoparticles, making it viable for industrial applications. Specific capacitance obtained using hollow nanostructures was nearly 70% higher than what could be achieved using solid structures (from the CV curves). The associated high cyclability can be directly linked to higher surface adsorption sites in hollow structures, along with enhanced channels for electrolyte ion adsorption-desorption. This would lead to efficient redox activities and ensure more than 90% coulombic efficiency, which makes the material useful for supercapacitors.

## Methods

There are many approaches for stabilizing hollow nanostructures. These include hard template based, soft template micro/miniemulsions, chemical etching, self-assembly, etc^66–68^. Direct synthesis of hollow nanostructures, at low temperature, is non-trivial and needs specially designed protocols. The synthesis processes used to obtain Cu_2_O and CuO are presented below:

### Synthesis of Cu_2_O materials

Analytical grade reagents, without further purification, were used as raw materials. For the synthesis of Cu_2_O hollow particles (Cu-HN), 2 g of Cu(NO_3_)_2_. 6H_2_O was initially taken in 80 mL of deionized (DI) water and stirred until a clear blue colored solution was obtained. The solution was then heated upto 70 °C and NaOH together with the reducing agent viz., dextrose, were added. The overall concentration of NaOH and dextrose in the solution were kept at 0.86 M and 0.16 M, respectively. On addition of NaOH and dextrose, solution’s color changed to dark blue that, after few minutes, turned to brick red. The solution was kept stirring for 60 min to ensure complete reaction. The obtained precipitate was subsequently centrifuged and washed three times using de-ionized (DI) water and ethanol. The final precipitate was dried at 80 °C for 12 h in vacuum.

To obtain solid Cu_2_O particles (Cu-OCT), experimental procedure similar to above was followed but with reagents concentrations reduced to half. 1 g of Cu(NO_3_)_2_.6H_2_O was mixed with 0.43 M of NaOH and 0.08 M of dextrose. Rest of the processes for initiating reaction, purification and annealing were similar.

Cu_2_O formation in both the cases can be written as:

9 $${\boldsymbol{C}}{{\boldsymbol{u}}}^{2+}+2{\boldsymbol{O}}{{\boldsymbol{H}}}^{-}\to {\boldsymbol{Cu}}{({\boldsymbol{OH}})}_{2}$$

10 $$2{\boldsymbol{Cu}}{({\boldsymbol{OH}})}_{2}+{{\boldsymbol{C}}}_{6}{{\boldsymbol{H}}}_{12}{{\boldsymbol{O}}}_{6}+{{\boldsymbol{H}}}_{2}{\boldsymbol{O}}\to {\boldsymbol{C}}{{\boldsymbol{u}}}_{2}{\boldsymbol{O}}+{{\boldsymbol{C}}}_{6}{{\boldsymbol{H}}}_{12}{{\boldsymbol{O}}}_{7}+3{{\boldsymbol{H}}}_{2}{\boldsymbol{O}}$$

### Synthesis of CuO hollow nanostructures

For the synthesis of hollow CuO, a strategy reported earlier by our group was used^43^.

### Characterization techniques

The phase formation of the synthesized materials was confirmed by analyzing the powder XRD profiles in the 2θ range 15–80° using the PAN Analytical diffractometer with Cu-Kα (λ = 0.15406 nm) as the incident wavelength. Scanning Electron Microscopy (SEM CARL ZEISS SUPRA 40) and transmission electron microscopy (TEMFEI-TECHNAI G220S-Twin operated at 200 kV) were used for undertaking the morphological analysis. Elemental analysis was done by analyzing EDAX spectra obtained using SEM CARL ZEISS SUPRA 40.Zeta potential and particle size measurements were performed using a Horiba Scientific Nano Particle Analyzer SZ-100.The BET surface area calculation and N_2_ adsorption-desorption data were obtained from Quantachrome Novatouch surface area analyzer. The artificial crystal morphological studies were performed using the SHAPE V7.3 software.

### Electrochemical measurements

Electrochemical measurements were performed using a three-electrode configuration having Ag/AgCl as the reference electrode and a platinum rod as the counter electrode. 2 M KOH aqueous solution was utilized as electrolyte. Slurries for both the Cu(HN) and Cu(OCT) were prepared separately. For the slurry preparation, 80 wt% of active material (Cu(HN)/Cu(OCT)), 10 wt% of activated carbon, and 10 wt% polyvinylidene fluoride were mixed using acetone as the mixing media. For homogenization, the mixture was stirred at 80 °C for 5 h. The slurry was drop casted onto a graphite sheet (1 cm × 1 cm) and electrochemical measurements such as: cyclic voltammetry, galvanostatic charge–discharge and electrochemical impedance spectroscopy were performed using the MetrohmAutolab (PGSTAT302N). EIS measurements were performed in the frequency range from 0.01 Hz to 100 kHz.

## Electronic supplementary material

Supplementary Information
