# Supplementary material for: Hollow nanostructures of metal oxides as next generation electrode materials for supercapacitors
Source: Sci Rep. 2018 Jan 22;8:1307. doi: 10.1038/s41598-018-19815-y (PMC5778045; doi:10.1038/s41598-018-19815-y)
Supplement: Supplementary file 1 — Supplementary Information [file 41598_2018_19815_MOESM1_ESM.pdf]

ELECTRONIC SUPPLEMENTARY INFORMATION

Hollow nanostructures of metal oxides as next generation electrode materials for supercapacitors

Vikas Sharma[1], Inderjeet Singh[2] and Amreesh Chandra[1,2]

[1]School of Nanoscience and Technology, Indian Institute of Technology Kharagpur, Kharagpur--721302, West Bengal, India

[2]Department of Physics, Indian Institute of Technology Kharagpur, Kharagpur--721302, West Bengal, India

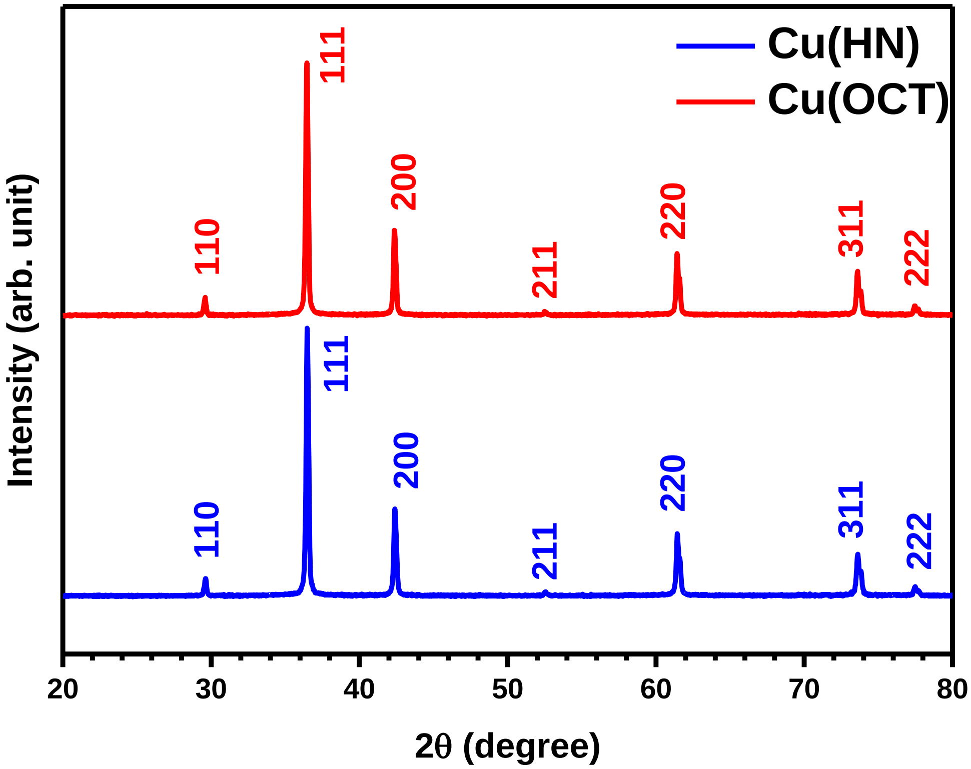

Fig.S1 XRD patterns of Cu_2O hollow (Cu(HN)) and solid octahedrons (Cu(OCT)).

Fig. S1 shows the XRD patterns of both the Cu(HN) and Cu(OCT). Both the patterns show clear phase formation with crystalline peaks representing [110], [111], [200], [211], [220], [311] and [222] planer diffractions. The phase of both the materials was confirmed by indexing the obtained peaks with JCPDS card no. 05-0667. Moreover, in the XRD patterns, no impurity peak was observed.

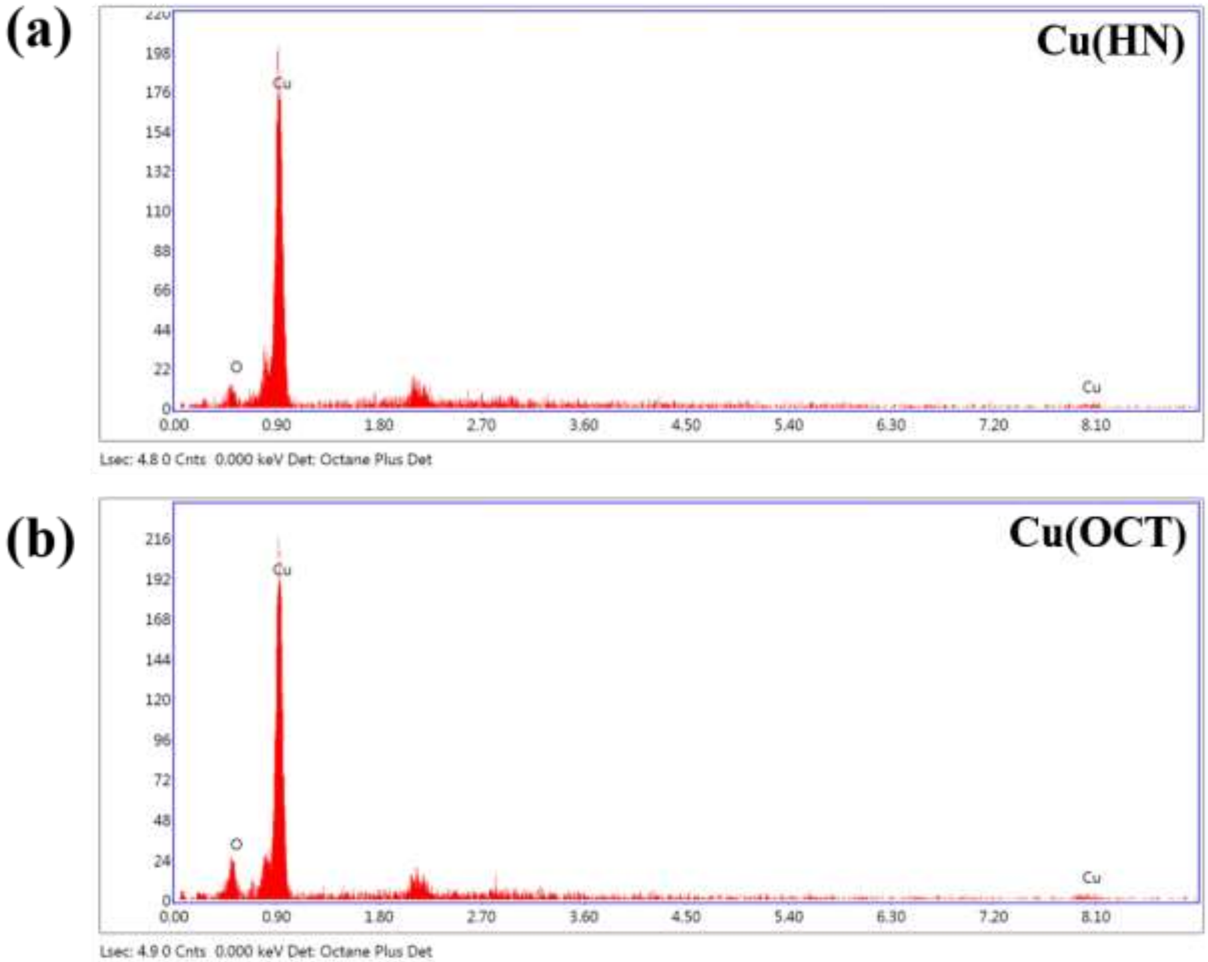

Fig.S2 EDX spectra of Cu(HN) and Cu(OCT).

Energy dispersive Analysis of X-rays (EDAX) measurements were performed for compositional analysis of the obtained Cu_2O materials. EDAX spectra of both Cu(HN) and Cu(OCT) are shown in Fig. S2(a, b). It estimated weight percentage of Cu:O was 96 : 4 in Cu(HN) and 91 : 9 in Cu(OCT). Table S1 shows the compositional information in detail obtained from EDAX spectra of Cu(HN) and Cu(OCT).

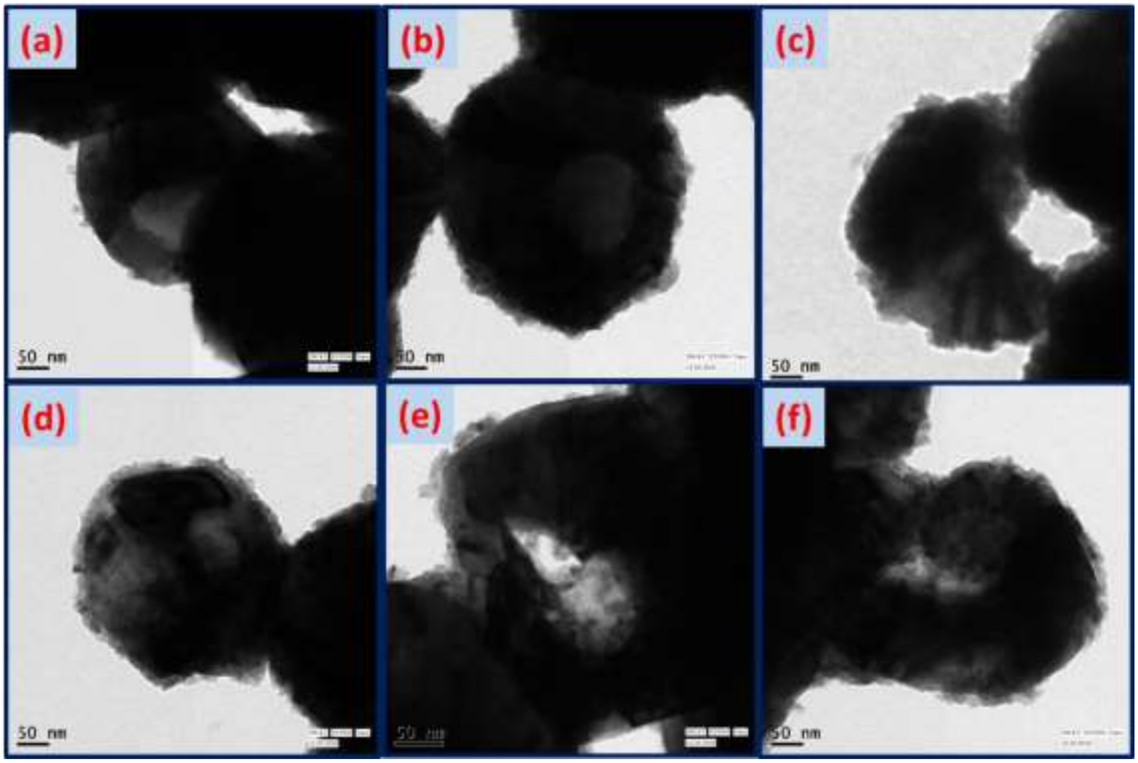

Fig. S3 (a-f) TEM images of Cu(HN) with hollow core identification.

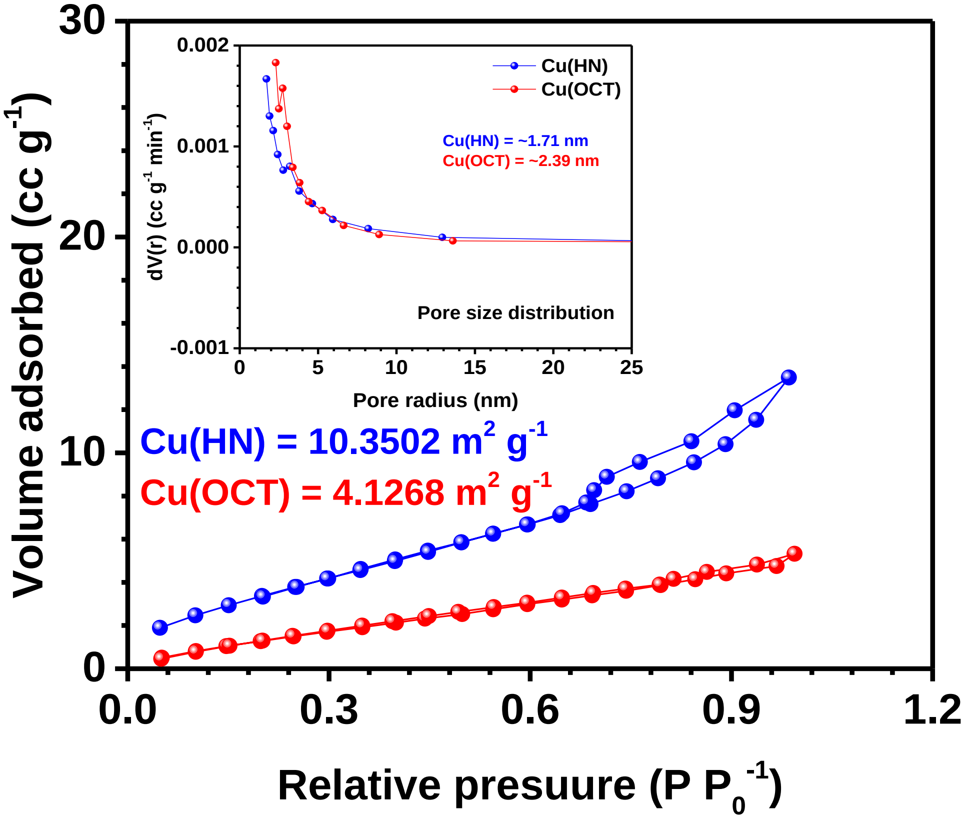

Fig. S4 N_2 adsorption desorption isotherm with surface area calculated.

Both the samples showed type IV isotherm with narrow hysteresis loop that is a typical characteristic of mesoporous materials. The BET surface area calculated was found to be ~10 m^2/g for Cu(HN) and ~4 m^2/g for Cu(OCT). This proves that more surface area is available for ion adsorption and desorption in case of Cu(HN). As the surface areas of both the materials are not tremendous, it further confirms that the materials have good pseudocapacitive nature and have large contribution in the overall specific capacitance achieved. Surface area of Cu(HN) is high which directs more ions inside and outside the cavity (both area contributes in adsorption-desorption process). Size of the hollow structures is also small in comparison to the octahedrons, which further contributes to the surface area of these materials in comparison to solid structures. Pore size distribution is also shown in the inset of the Fig. S4 showing the pore radius for

Cu(HN) and Cu(OCT) to be ~1.71 nm and ~2.39 nm, respectively. It is well known fact that smaller pore radius is considered as ideal for an electrode material in supercapacitors. Because of the higher specific surface area and narrower pore radius distribution, proper channels for ion diffusion are generated in Cu(HN) which leads to high charge storage capacity in these structures.

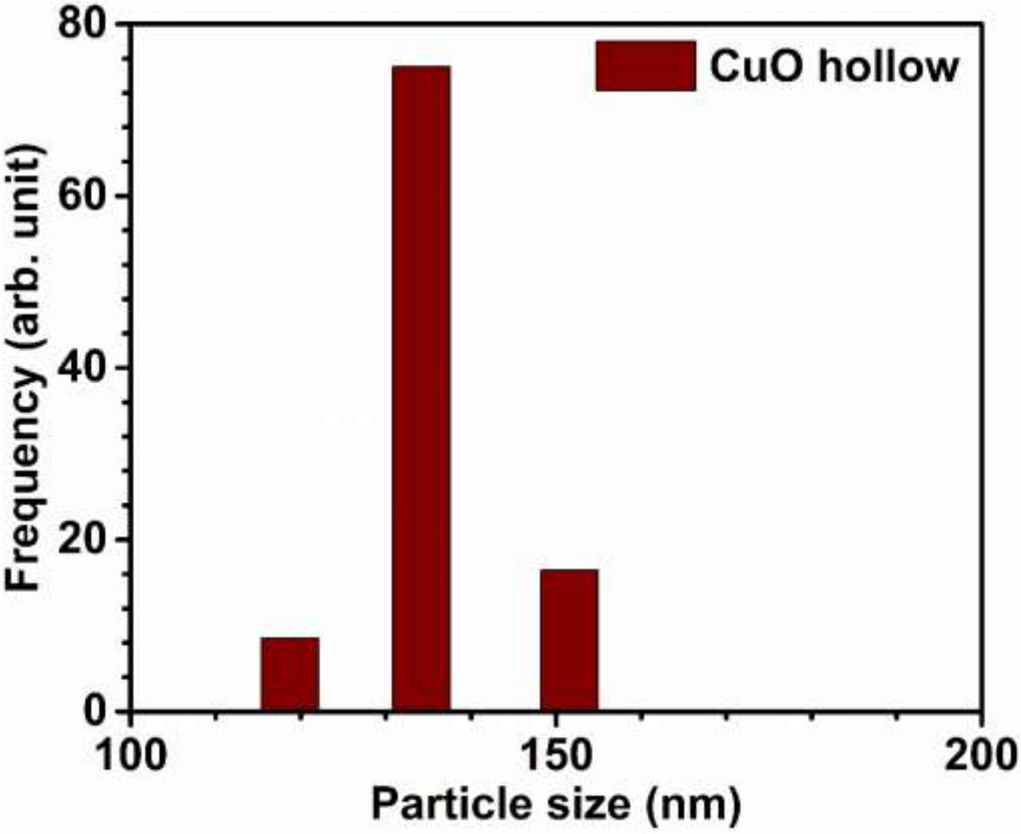

Fig.S5 Particle size distribution of CuO hollow nanostructures.

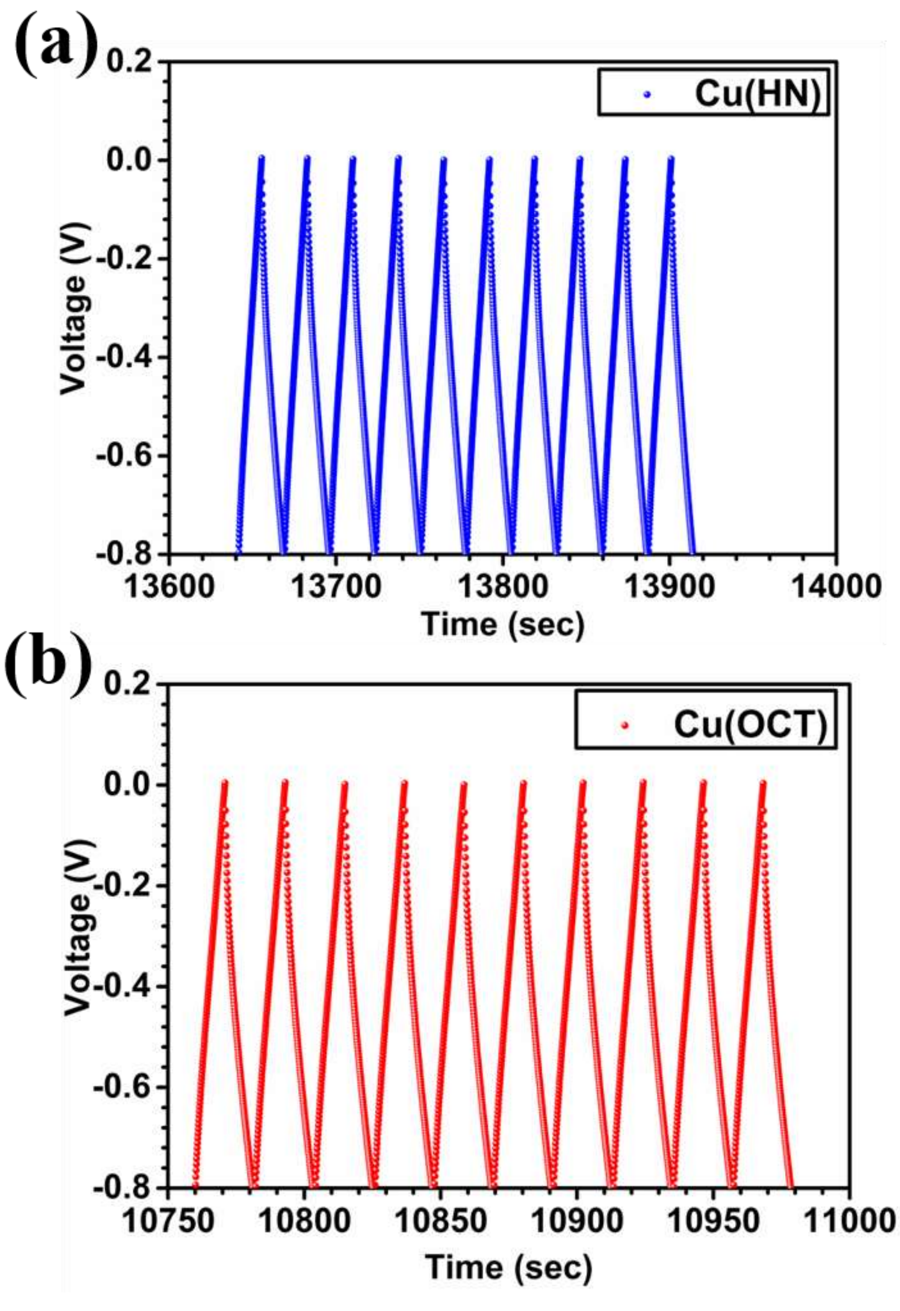

Fig.S6 Cycling curves of (a) Cu(HN) and (b) Cu(OCT).

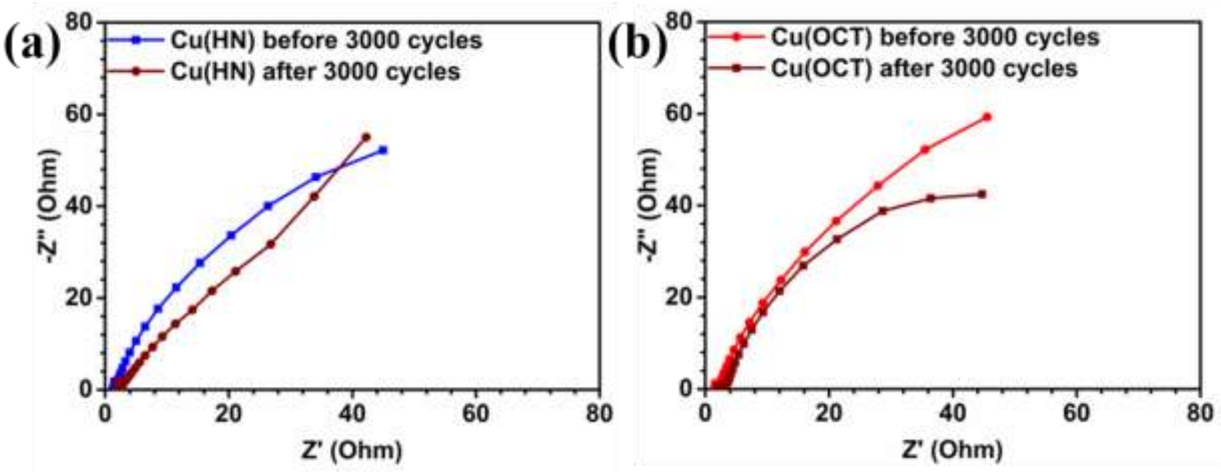

Fig.S7 EIS plots of (a) Cu(HN) and (b) Cu(OCT) before and after 3000 cycles.

CuO hollow nanostructures electrochemical performance

For giving the further evidence in support of the claim that the hollow structures have superior electrochemical properties than the solid structures, we have tested CuO hollow nanostructures as electrode material in 3 M KOH as electrolyte in three electrode cell configuration. It is already reported that the surface area of hollow nanostructures of CuO is nearly four times than the solid CuO particles. CV, CD and EIS measurements were formed for these CuO hollow nanostructures as working electrodes using similar configuration as used for Cu(HN) and Cu(OCT).

Fig. S8(a, b) shows the CV and CD curves of CuO hollow nanostructures. The stable voltage window was found to be -0.65 to 0.55 V (~1.2 V) and the CV curves in this range showed clear redox peaks, which are the characteristic peaks of CuO. The values of specific capacitance are listed in Table S4 and the maximum specific capacitance was found to be 140 F g^{-1} at scan rate of 10 mV s^{-1}. From the CD curves, the maximum specific capacitance was found to 94 F g^{-1} at 3 A g^{-1} current density with capacitance retention of ~ 68 % when the current density was increased from 3 A g^{-1} to 10 A g^{-1} (Fig. S8(a,b)). One special advantage of these CuO hollow nanostructures was found to be the large stable voltage window of 1.2 V. This is much higher than the reported voltage windows for various CuO morphologies till now.

Fig. S8(c) shows the EIS curve of the CuO hollow nanostructures as electrode, which shows that the ESR value was 1.48 Ω in the first cycle. The linear portion in the low frequency region proves the usefulness of CuO hollow nanostructures as capacitive material. The cyclic stability for the electrode was tested for 2000 subsequent cycles at 3 A g^{-1}. The material was found to retain 87.23 % of the original specific capacitance even after 3000 subsequent cycles as

shown in Fig. S8(d). The decrease in the specific capacitance could be attributed to the increase in the ESR value from 1.48 Ω to 1.78 Ω, as per the EIS curve shown in Fig. S8(c). The variation of specific capacitance with scan rates and current densities is shown in Fig. S9(a,b).

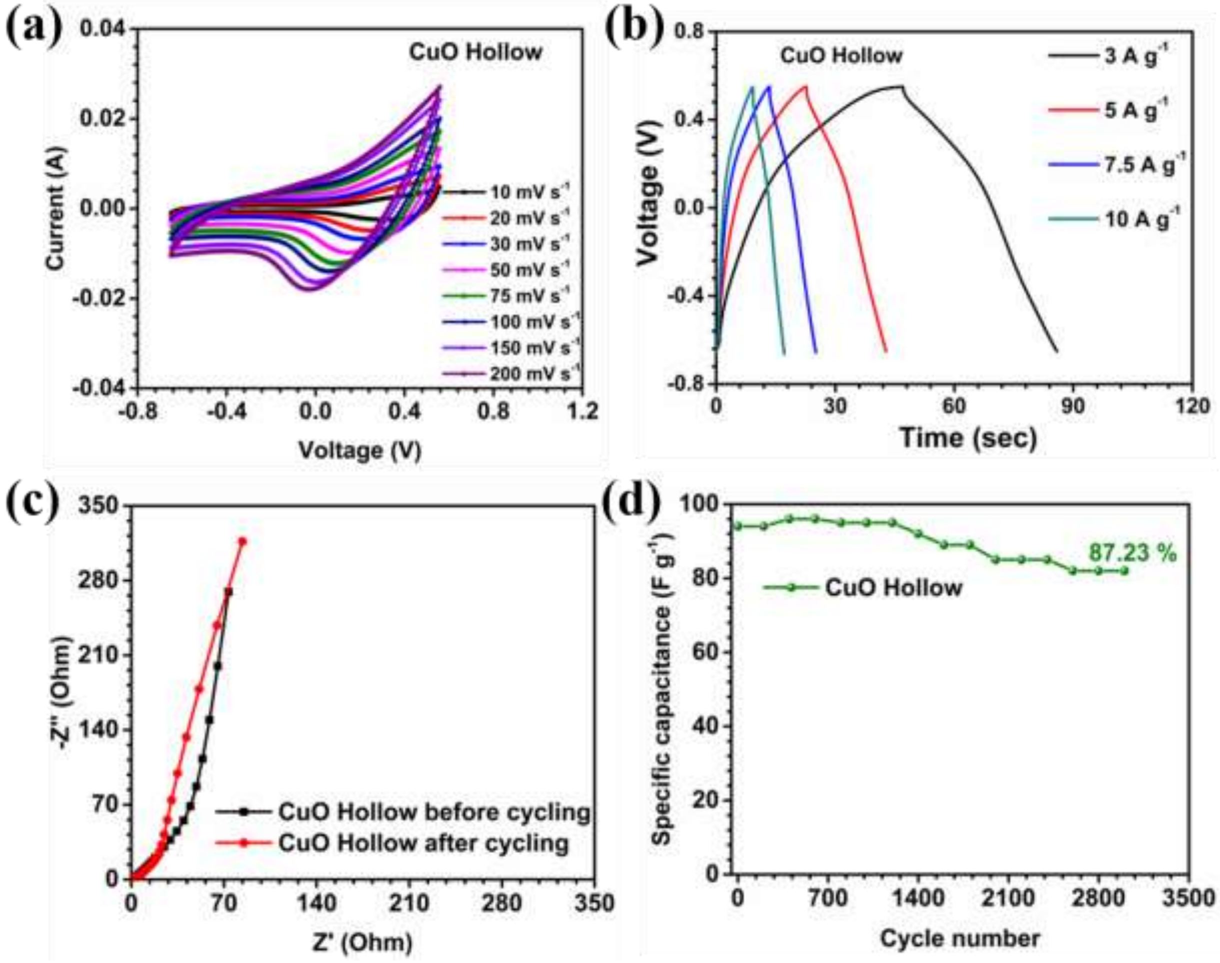

Fig.S8 (a) CV curves at different scan rates, (b) CD curves at different current densities, (c) EIS plots before and after cycling and (d) variation of specific capacitance with cycling at 3 A g^{-1} for CuO Hollow nanostructures.

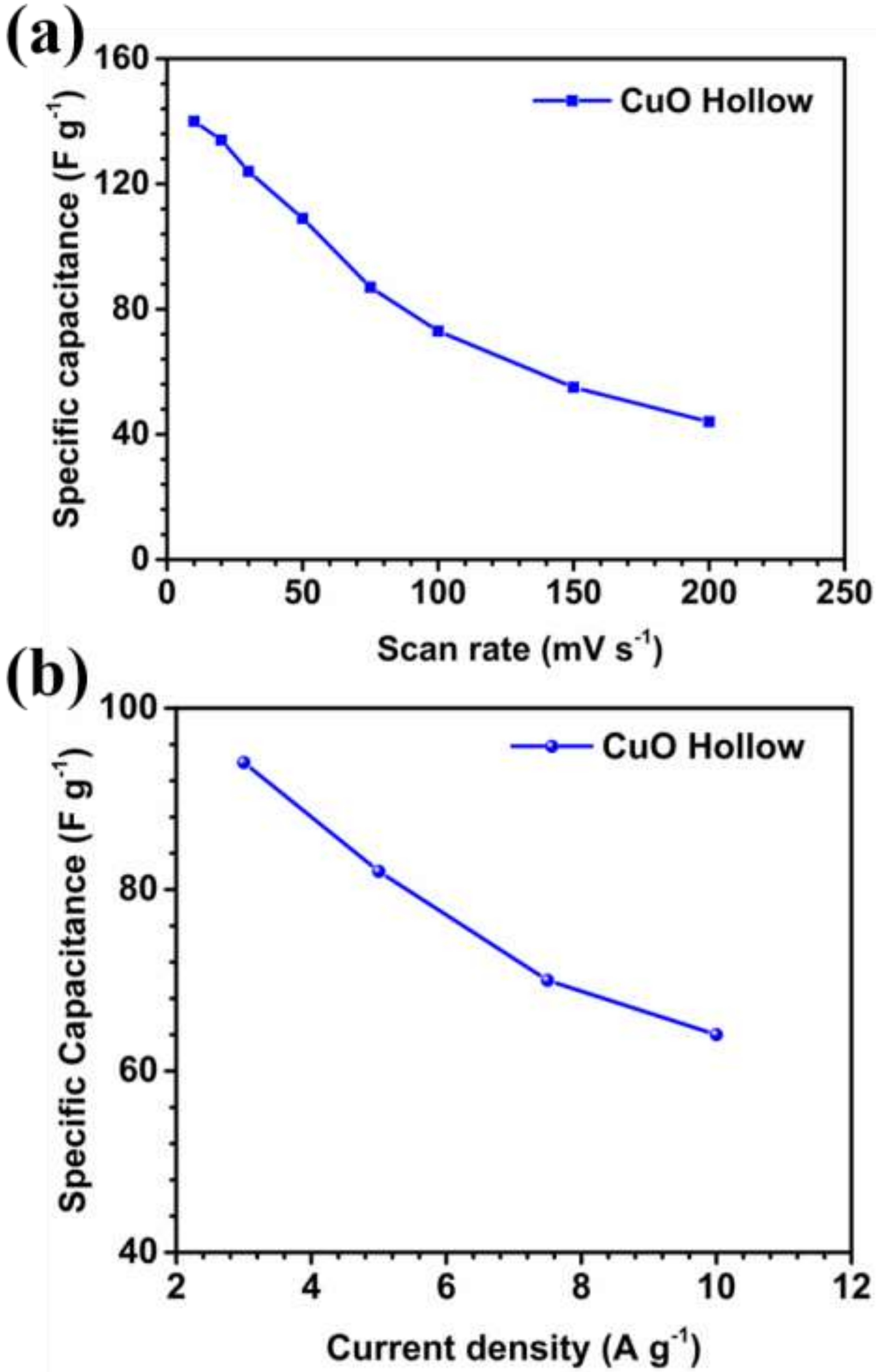

Fig.S9 Variation of specific capacitance of CuO Hollow with (a) scan rate and (b) current density.

Sample name	Element	Weight %	Atomic %	Net Int.	Error %	K ratio	Z	R	A	F
Cu(HN)	O K	4.38	15.40	30.00	23.01	0.04	1.37	0.86	0.6	1
	CuL	95.62	84.60	444.53	4.63	0.92	0.98	1.01	0.98	1
Cu(OCT)	O K	8.73	27.53	68.33	14.46	0.07	1.34	0.86	0.61	1
	CuL	91.27	72.47	464.23	4.72	0.84	0.97	1.02	0.96	1

Table S1 Summarized EDAX results for Cu(HN) and Cu(OCT).

Scan Rate (mV s^{-1})	Cu(HN) Specific Capacitance (F g^{-1})	Cu(OCT) Specific Capacitance (F g^{-1})
10	164	97
20	150	92
30	146	89
50	144	89
75	144	86
100	143	84
150	142	80
200	139	77

Table S2 Specific capacitance values at different scan rates for Cu(HN) and Cu(OCT).

Current Density ($A\ g^{-1}$)	Cu(HN) Specific Capacitance ($F\ g^{-1}$)	Cu(OCT) Specific Capacitance ($F\ g^{-1}$)
**1**	**144**	**105**
**2**	**115**	**90**
**3**	**105**	**79**
**5**	**87**	**67**
**7.5**	**79**	**56**
**10**	**71**	**50**

Table S3 Specific capacitance values at different current densities for Cu(HN) and Cu(OCT).

Scan Rate ($mV\ s^{-1}$)	CuO Hollow Specific Capacitance ($F\ g^{-1}$)	Current density ($A\ g^{-1}$)	CuO Hollow Specific Capacitance ($F\ g^{-1}$)
**10**	**140**	**3**	**94**
**20**	**134**	**5**	**82**
**30**	**124**	**7.5**	**70**
**50**	**109**	**10**	**64**
**75**	**87**		
**100**	**73**		
**150**	**55**		
**200**	**44**		

Table S4 Specific capacitance values at different scan rates and current densities for CuO Hollow.
